# A Facile Method for Synthesizing Cobalt Oxide Nanoparticles to Create a Highly Sensitive Non-Enzyme Glucose Sensor

**DOI:** 10.3390/bios15040235

**Published:** 2025-04-07

**Authors:** Zhanar K. Kalkozova, Ulpan A. Balgimbayeva, Maratbek T. Gabdullin, Lesya V. Gritsenko, Guoquan Suo, Khabibulla A. Abdullin

**Affiliations:** 1 Institute of Applied Science & Information Technology, Shashkin Str. 40-48, Almaty 050040, Kazakhstan; zhanar.kalkozova@kaznu.edu.kz; 2 National Nanotechnology Laboratory of Open Type (NNLOT), Al-Farabi Kazakh National University, Al-Farabi Ave., 71, Almaty 050040, Kazakhstan; 3 School of Materials Science and Green Technology, Kazakh-British Technical University, Tole bi Street, 59, Almaty 050000, Kazakhstan; u.balgimbaeva@kbtu.kz (U.A.B.); m.gabdullin@kbtu.kz (M.T.G.); 4 General Physics Department, Institute of Energy and Mechanical Engineering, Satbayev University, Satpayev Str., 22, Almaty 050013, Kazakhstan; 5 School of Materials Science and Engineering, Shaanxi University of Science & Technology, Xi’an 710021, China; suoguoquan@sust.edu.cn

**Keywords:** cobalt hydroxycarbonate, chemical bath deposition, enzyme free, electrochemical sensor, glucose

## Abstract

In this study, an electrochemical non-enzymatic glucose sensor based on cobalt oxide was developed using a simple chemical bath deposition method. The as-synthesized material exhibited no significant sensitivity; the latter emerged only after subsequent electrochemical activation. To the best of our knowledge, this is the first report demonstrating the successful application of electrochemical activation to achieve enhanced sensitivity. An X-ray diffraction analysis confirmed that a single-phase Co_2_(OH)_2_(CO_3_) material was obtained immediately after synthesis, which was subsequently transformed into Co_3_O_4_ nanoparticles during electrochemical activation. SEM and TEM analyses revealed that the synthesized particles initially exhibited a nanorod structure, which evolved into a highly dispersed form after activation. The non-enzymatic glucose sensor based on the electrochemically activated material demonstrated excellent glucose sensitivity of 33,245 µA mM^−1^ cm^−2^ within the linear range of 0–0.5 mM, with a detection limit (LOD) of 5 µM. The starting material remained stable for over 12 months under ambient storage conditions and regained its high sensitivity following electrochemical activation.

## 1. Introduction

The development of enzyme-free glucose sensors is highly relevant due to the growing prevalence of diseases caused by impaired glucose metabolism, such as diabetes, and the need to enhance health monitoring and maintain overall well-being. Traditional glucose monitoring methods rely on enzyme-based sensors, which are widely used in medical practice. However, enzyme-based sensors have several disadvantages, including susceptibility to degradation, sensitivity to external factors and interfering substances, a short lifespan, and high cost. As a result, the development of materials for non-enzymatic glucose sensors to overcome these limitations is of significant practical importance. Enzyme-free sensors offer stable and long-lasting performance while reducing production and operating costs [1,2,3], making them a promising solution for developing affordable and effective glucose monitoring systems. Various nanomaterials and nanocomposites exhibit enhanced electrochemical properties due to their high specific surface area, advanced surface morphology, and presence of catalytically active surface centers [4,5,6].

Enzyme-free glucose sensors based on noble metals (Pt, Au, Ag), transition metals (Co, Ni, Zn, Cu, Fe, etc.), and their oxides have demonstrated high sensitivity and stability and have been extensively developed in recent years [1,2,3,4,5,6,7,8,9,10,11,12,13]. Composites incorporating polymers and carbon materials combined with metal and oxide nanoparticles [14,15,16,17] have also shown high efficiency in the development of enzyme-free sensors. Various three-component nanocomposites, such as Cu-Zn-Mn, Pd-Cu-Au, Au-Cu-Ni, and their corresponding oxides, exhibit a synergistic effect, enhanced functionality, greater stability, and higher activity compared to monodisperse and bimetallic composites in glucose detection [13].

For example, a composite based on carbon fiber and NiO-CuO demonstrated a linear range from 1.32 µM to 0.570 mM, with an exceptionally high sensitivity of 70,000 µA mM^−1^ cm^−2^ and a detection limit of 0.40 µM [18]. Copper oxide sensors have been extensively studied [19,20,21,22,23,24,25] and are known for their high sensitivity, low detection limits, excellent selectivity, and resistance to external influences. Arrays of ZnO nanorods, used as components in composite sensors [25,26,27], have also demonstrated significant potential for application in glucose sensing.

Cobalt compounds, such as cobalt phosphate nanoparticles [28], Co(OH)_2_ hydroxide nanosheets [29], and Co_3_O_4_ oxide [30], are also promising materials for developing glucose sensors. For instance, an electrochemical non-enzymatic glucose sensor based on cobalt oxide was fabricated through laser spraying of a hydrogel containing cobalt precursors [30]. This sensor achieved a sensitivity of 187.129 μA mM^−1^ cm^−2^ within the range of 1–30 µM and a low limit of detection (LOD) of 0.10 µM.

Thus, the synthesis of new materials for electrochemical glucose sensors is a highly important practical task, with a broad range of materials showing promise for the enzyme-free development of glucose sensors. Equally important is the development of simple and cost-effective synthesis methods capable of producing sensor materials with high sensitivity, stability, and significant application potential. In mass production, the efficiency of the synthesis process is critical, emphasizing the need for simple technological methods, low energy consumption, and inexpensive precursors. In composite materials, the inclusion of multiple components with differing properties can result in a synergistic effect for glucose detection. However, optimizing the process to achieve an ideal sensor structure is significantly more complex compared to that for single-component sensor materials.

This article presents a cobalt oxide sensor for the highly sensitive determination of glucose levels, fabricated using a simple chemical bath deposition method followed by electrochemical activation. To the best of our knowledge, this is the first application of electrochemical activation to the as-synthesized material to enhance its sensitivity. The synthesis and activation processes were conducted at temperatures below 90 °C using widely available and cost-effective precursors. The sensor exhibits high sensitivity within a linear range of 0–0.5 mM and a low detection limit.

## 2. Materials and Methods

### 2.1. Materials

Cobalt(II) nitrate hexahydrate Co(NO_3_)_2_∙6H_2_O, urea, and anhydrous glucose were purchased from Sigma Aldrich (St. Louis, MO, USA). Acetylene black was purchased from Fuzhou Yihuan Carbon Ltd. (Fuzhou, China). An AQUA-MAX-Ultra 370 Series ultrapure water purification system (YL Instrument Co., Anyang, Republic of Korea) was used to obtain MilliQ water (18.2 MOhm·cm) for the synthesis of samples and to carry out electrochemical measurements.

### 2.2. Synthesis

The chemical bath deposition method was used to synthesize powdered samples. Quantities of 6 g of cobalt(II) nitrate hexahydrate and 0.5 g of acetylene black were added to a glass beaker containing 300 mL of water. The mixture was stirred on a magnetic stirrer until the cobalt nitrate was completely dissolved. The beaker was then placed in an ultrasonic bath for 2 h to disperse the acetylene black. Seven grams of urea was dissolved in 50 mL of water and added to the beaker containing cobalt nitrate, and the total volume of the solution was adjusted to 400 mL with water. The synthesis was performed in a water bath with vigorous stirring at 90 °C for 4 h. After synthesis, the precipitates were washed several times with deionized water by centrifugation and dried in air at 90 °C overnight. The resulting sample was subsequently used for electrochemical measurements.

### 2.3. Electrochemical Measurements

Electrochemical measurements were performed in a conventional electrochemical cell using a three-electrode system and 0.1 M KOH aqueous solution. The sample, prepared as a suspension of the synthesized powder in ethanol, was applied to a thoroughly cleaned glassy carbon working electrode with a diameter of 2 mm. After drying, a layer of the adsorbed sample remained on the end surface of the glassy carbon working electrode. A platinum wire served as the counter electrode, and an Ag/AgCl electrode was used as the reference electrode. An aqueous solution of potassium hydroxide was employed as the electrolyte. To determine the sensitivity of the sensor, glucose was dissolved in 0.1 M KOH and subsequently introduced in defined quantities into the electrochemical cell electrolyte under continuous stirring. A Corrtest CS2350 potentiostat (Wuhan Corrtest Instrument Corp., Wuhan, China) was used to record cyclic voltammetry curves and perform impedance spectroscopy measurements.

Modification of the sensory properties of the material was carried out by electrochemical activation, which was performed using cyclic voltammetry (CV) measurements in the range of potential from −0.6 to 0.8 V vs. Ag/AgCl.

The electrochemical activation rate was low in the 0.1 M KOH electrolyte. Therefore, electrochemical activation was performed in a 3.5 M KOH electrolyte, chosen as a compromise between the activation rate and reagent consumption. Typically, the CV curves measured at a potential sweep rate of 100 mV s^−1^ within a range of −600 mV to +750 mV relative to the Ag/AgCl reference electrode stabilized in the 3.5 M KOH electrolyte after 40–50 cycles.

### 2.4. Characterization Techniques

XRD measurements to determine the phase structure of the samples were performed using a MiniFlex X-ray diffractometer (Rigaku, Tokyo, Japan) operating with CuKα radiation at a wavelength of 1.5418 Å. Scanning electron microscopy (SEM) studies to characterize the morphology of the samples were performed using a Quanta 200 microscope (FEI, Hillsboro, OR, USA). A NEXSA X-ray Photoelectron Spectrometer (Thermo Scientific, Waltham, MA, USA) was used to collect XPS spectra.

## 3. Results

### 3.1. Morphology and Structure

**Figure 1:**
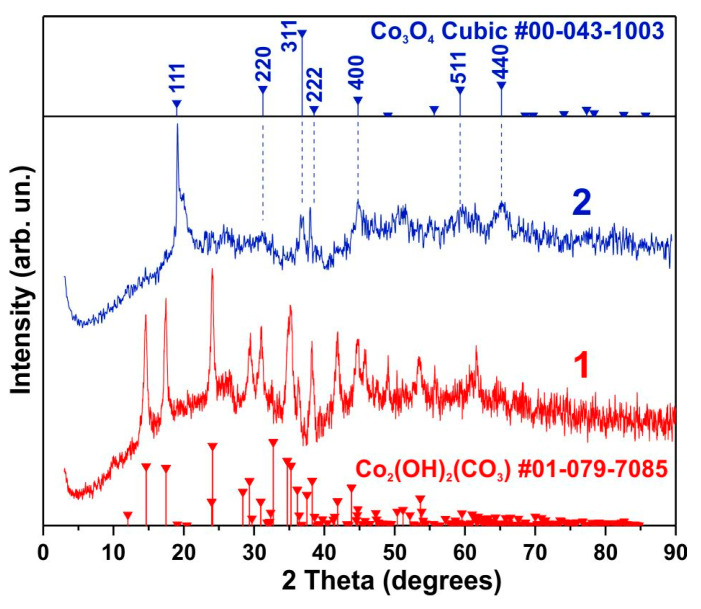
XRD data for the sample immediately after synthesis (**1**) and after electrochemical activation (**2**).

Figure 1 presents the XRD results for the synthesized samples (curve 1) and the samples after electrochemical activation (curve 2). All observed reflections in the initial sample can be attributed to the phase of cobalt hydroxycarbonate, Co_2_(OH)_2_(CO_3_), which corresponds to PDF card No. 01-079-7085. The initial samples do not exhibit sensitivity to glucose; such sensitivity is observed only after electrochemical activation, which leads to the formation of a new cobalt oxide phase. As shown in Figure 1 (curve 2), the XRD pattern of the sample after activation aligns well with the standard Co_3_O_4_ phase (PDF card No. 00-043-1003). The intense reflections of the (111) and (222) planes indicate the presence of a certain number of Co_3_O_4_ particles elongated along the [111] direction. Simultaneously, a significant broadening of these and other observed reflections corresponding to the Co_3_O_4_ phase is evident.

### 3.2. SEM and TEM Results

**Figure 2:**
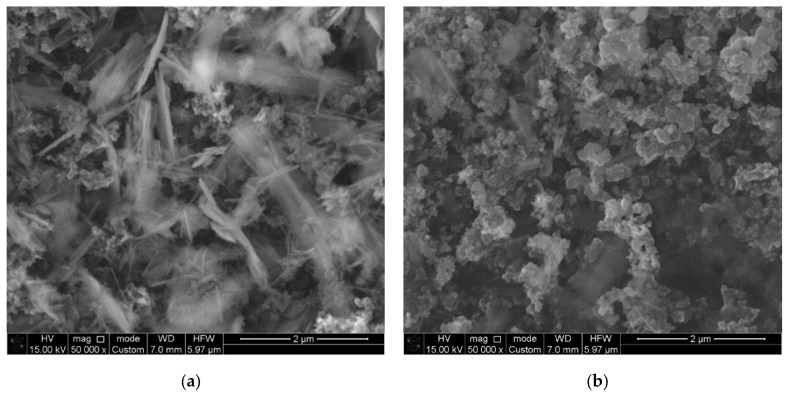
SEM images of the sample after synthesis (**a**) and after electrochemical activation (**b**).

Figure 2 presents the SEM results, illustrating the morphology of the samples both immediately after synthesis (Figure 2a) and after subsequent electrochemical activation (Figure 2b). As shown in Figure 2a, the synthesized cobalt hydroxycarbonate particles exhibit a nanorod-like morphology with a length of approximately 1 μm and a diameter of about 20–40 nm. Compact particles with characteristic sizes of 50 nm are identified as soot particles. Electrochemical activation induces significant morphological changes, leading to the transformation of the nanorods into compact nanoparticles, as observed in Figure 2b.

**Figure 3:**
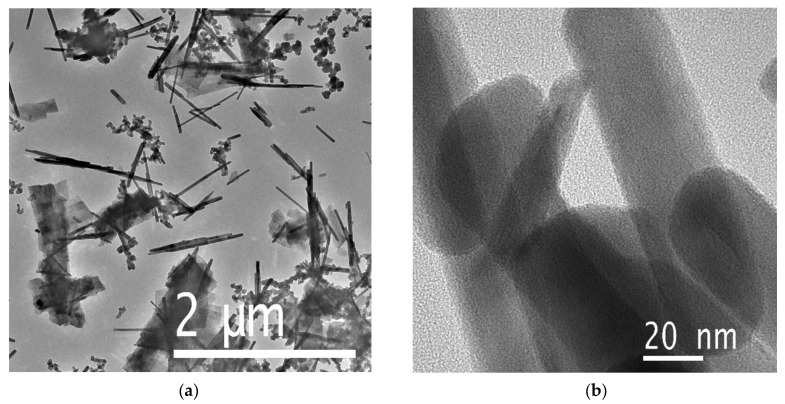
TEM images of the as-synthesized sample: (**a**) low-resolution and (**b**) high-resolution images.

**Figure 4:**
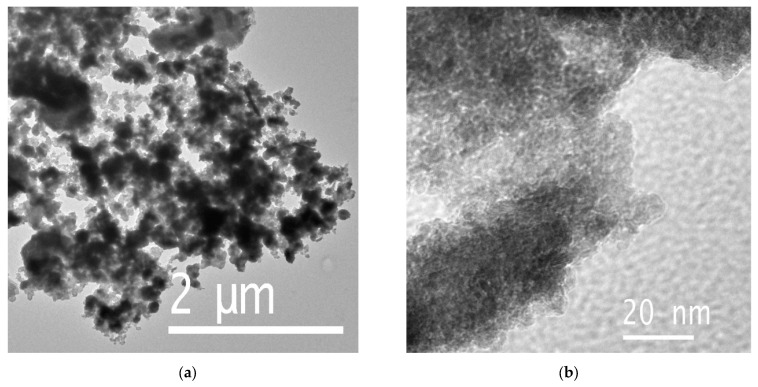
TEM images of the sample after electrochemical activation: (**a**) low-resolution and (**b**) high-resolution images.

The TEM analysis of the sample morphology, presented in Figure 3 and Figure 4, corroborates the SEM findings. The as-synthesized material primarily consists of rod-like structures, with a fraction present in the form of thin plates (Figure 3). Consistent with the SEM observations, soot particles with characteristic sizes of approximately 50 nm are also detected. Prior to activation, the synthesized rods exhibit a homogeneous structure with uniform electron density, indicating a monocrystalline nature.

Following activation, the nanorods and plates are no longer observed; instead, the material transforms into a highly dispersed polycrystalline form, with particle sizes of approximately 10 nm (Figure 4). These results demonstrate that the morphology and phase composition of the material undergo substantial changes during activation.

### 3.3. XPS Spectra

**Figure 5:**
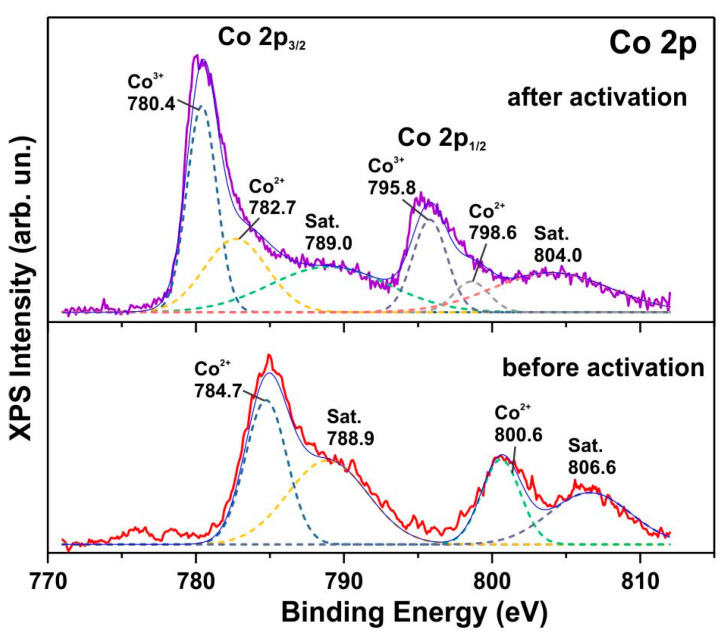
High-resolution X-ray spectra of Co 2p for both the synthesized sample and the sample after activation. The dotted lines demonstrate the decomposition of the XPS spectra into components that can be attributed to Co^3+^, Co^2+^ and satellite lines (Sat.).

The pronounced effect of electrochemical activation on the structure of the initial cobalt hydroxycarbonate phase was confirmed by XPS analysis. Figure 5 presents the high-resolution Co 2p XPS spectra for both the as-synthesized and electrochemically activated materials. The XPS spectrum of the initial sample exhibits peaks at binding energies of 784.7 eV (Co 2p_3/2_) and 800.6 eV (Co 2p_1/2_), along with satellite peaks at 788.9 eV and 806.6 eV. These binding energies are notably high, exceeding those of the corresponding Co^2+^ peaks in Co(OH)_2_ (~781.5 eV and ~797 eV, respectively) [31] and approaching the characteristic Co^2+^ peak positions in CoCO_3_ [32]. This indicates that cobalt in the initial sample is exclusively in the Co^2+^ oxidation state. Following electrochemical activation, the XPS cobalt spectrum becomes characteristic of the Co_3_O_4_ phase. The Co 2p_3/2_ region then contains peaks at 780.4 eV and 782.7 eV, which can be attributed to Co^3+^ and Co^2+^ states, respectively, within the Co_3_O_4_ structure [33], along with a satellite peak at 789 eV. The Co 2p_1/2_ band can also be interpreted as a superposition of two components corresponding to Co^3+^ and Co^2+^ states in the Co_3_O_4_ phase.

**Figure 6:**
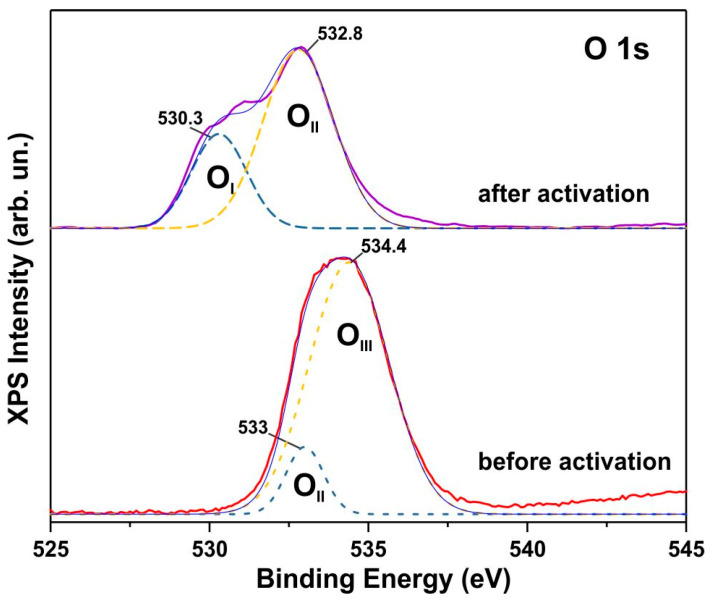
High-resolution O 1s XPS spectra of the as-synthesized and activated samples. The dotted lines show the decomposition of the XPS spectra into components OI, OII and OIII.

Three peaks with progressively increasing binding energies are distinguishable in the O 1s XPS spectra (Figure 6): the OI peak at 530.3 eV (low binding energy), the OII peak in the range of 531.8–533.0 eV (moderate binding energy), and the OIII peak at 534.4 eV (high binding energy). Based on literature data [34], the OI band at 530.3 eV is attributed to lattice oxygen anions (O^2−^) in Co_3_O_4_. Peaks within the 531.8–533.0 eV range (OII) are associated with O–C and O–H bonds, adsorbed OH^−^ and CO_3_^2−^ groups [34], or oxygen weakly bound to the lattice, such as in vacancy defects. The high-energy OIII peak at 534.4 eV is unequivocally attributed to adsorbed water. The as-synthesized material exhibits a broad O 1s XPS band that can be deconvoluted into two components. The O 1s peak at 533 eV (OII) corresponds to O–C and O–H bonds, while the peak at 534.4 eV (OIII) is attributed to adsorbed water, consistent with the expected O 1s XPS spectrum of the Co_2_(OH)_2_(CO_3_) phase. Upon activation, an additional OI band appears, which is characteristic of oxygen anions (O^2−^) in the metal oxide lattice [34]. This provides further evidence of Co_3_O_4_ phase formation.

**Figure 7:**
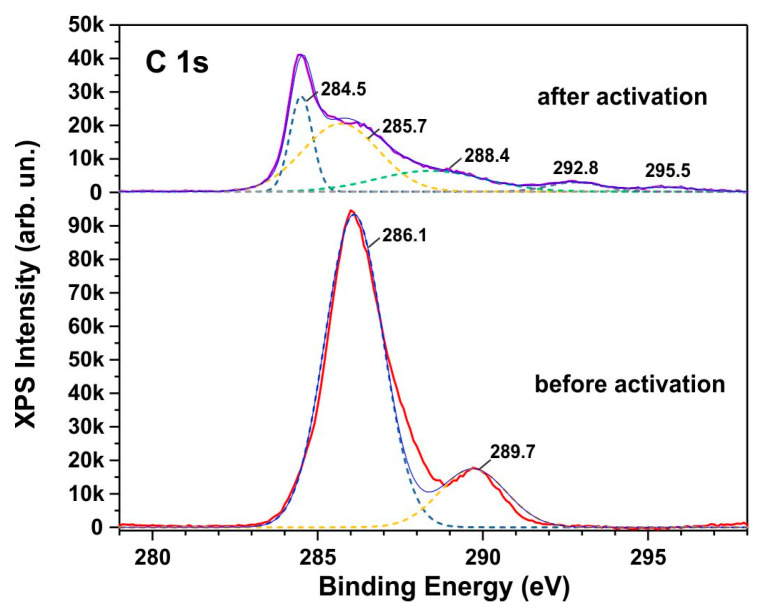
High-resolution C 1s XPS spectra for as-synthesized and activated samples. The dotted lines demonstrate the decomposition of the XPS spectra into individual components.

In the as-synthesized sample, where XRD analysis confirms the dominance of the Co_2_(OH)_2_(CO_3_) phase, two distinct bands are observed in the C 1s XPS spectra (Figure 7). The C 1s peak at 286.1 eV is attributed to C–O bonds in Co_2_(OH)_2_(CO_3_), while the peak at 289.7 eV corresponds to CO_3_^2−^ radicals [35]. The similarity between the C 1s XPS spectra of the activated samples and those characteristic of Co_3_O_4_ [36] further supports the structural modification revealed by XPS analysis of cobalt and oxygen. Thus, XRD, SEM, TEM, and XPS analyses collectively demonstrate the effect of electrochemical activation in transforming the Co_2_(OH)_2_(CO_3_) structure into Co_3_O_4_.

### 3.4. Electrochemical Glucose Sensing

It should be noted that since electrochemical activation initiated the electrochemical oxidation of Co_2_(OH)_2_(CO_3_) and the formation of a new Co_3_O_4_ phase, the adhesion of the sample was disrupted due to changes in its specific volume. As a result, part of the sample lost contact with the glassy carbon working electrode, leading to significant current surges during the CV measurements. To eliminate this effect, the end surface of the glassy carbon electrode with the sample was pressed against a porous ceramic plate, preventing the sample from peeling off.

**Figure 8:**
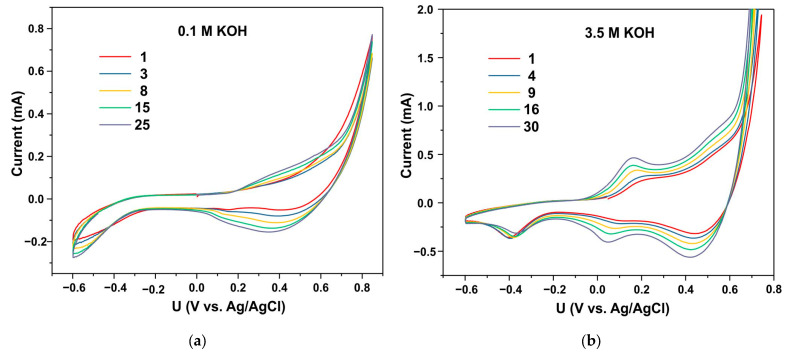
The modification of CV curves for the starting material in both 0.1 M (**a**) and 3.5 M KOH (**b**) electrolytes. The numbers on the curves represent the numbers of CV cycles.

The CV curves in Figure 8 demonstrate a modification of the electrochemical characteristics of the starting material during the transformation of the Co_2_(OH)_2_(CO_3_) phase into Co_3_O_4_ in both 0.1 M and 3.5 M electrolytes. However, in the latter case, the transformation occurs at a significantly higher rate, with CV curve stabilization achieved after approximately 30–40 cycles.

The synthesized material was tested for its glucose sensitivity. It was found that the starting material did not exhibit glucose sensitivity immediately after synthesis. Electrochemical activation of the material significantly enhanced its electrochemical conductivity (Figure 8). Anodic and cathodic peaks appear on the CV curves, corresponding to the redox transitions of the Co^2+^ ↔ Co^3+^ and Co^3+^ ↔ Co^4+^ centers [2,37]. The presence of redox peaks indicates that the initial phase formed during synthesis transforms into a cobalt oxide phase as a result of electrochemical activation. Following activation, the material was thoroughly rinsed with water. Subsequent CV measurements in a 0.1 M KOH electrolyte demonstrated that high conductivity was retained.

**Figure 9:**
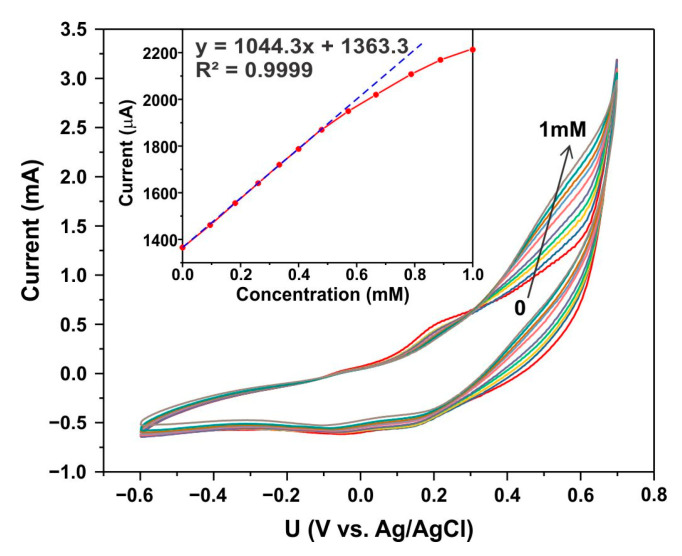
Cyclic voltammetry curves demonstrating a sequential increase in glucose concentration from 0 to 1 mM, recorded at a scan rate of 100 mV s^−1^. The inset displays the dependence of the current at a potential of 0.6 V vs. Ag/AgCl on the glucose concentration.

The activated sample exhibited a highly sensitive response to the presence of glucose. Figure 9 illustrates the transformation of CV curves with a stepwise increase in the glucose concentration from 0 to 1 mM. The inset in Figure 9 shows the relationship between the current at a potential of 0.6 V vs. Ag/AgCl and the glucose concentration. In the linear range, the sensitivity was determined to be 33,245 μA mM cm^−2^, with a detection limit (LOD) of 5 µM. It is worth noting that these sensitivity and LOD parameters were achieved with a sample weighing less than 0.1 mg. The linear sensitivity range extends up to a glucose concentration of 0.5 mM.

**Figure 10:**
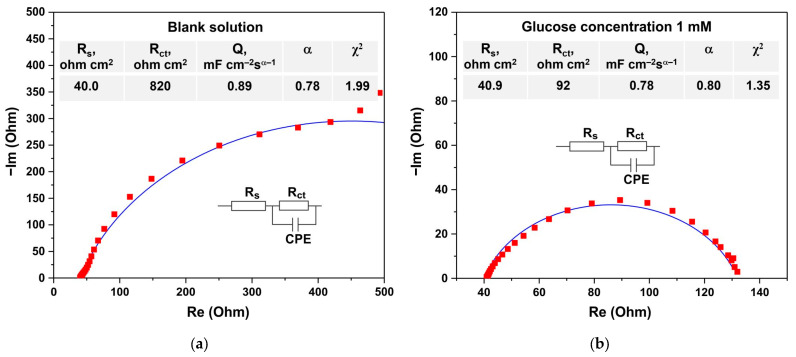
Nyquist plots recorded at potentials of 0.5 V vs. Ag/AgCl in a blank electrolyte (**a**) and in an electrolyte containing 1 mM glucose (**b**). Squares represent the experimental data points, while the lines correspond to the fitting based on the proposed equivalent circuit.

Figure 10 presents the results of electrochemical impedance spectroscopy (EIS) measurements conducted at a potential of 0.5 V vs. Ag/AgCl. The equivalent circuit corresponding to the Nyquist plot consists of the solution resistance (R_s_), constant phase element (CPE) $Z_{CPE}=\frac{1}{Q{\left(i\omega \right)}^{\alpha }}$, and charge transfer resistance (R_ct_). The parameters of the equivalent circuit, obtained by fitting spectroscopy data, are shown in Figure 10a,b. The χ^2^ coefficient, which quantifies the discrepancy between the experimental and modeled data, is also provided. The χ^2^ value was calculated using Equation (1):

(1) $$\chi^{2}=\sum_{i=1}^{N}{\left(\frac{Z_{exp,i}^{Re}{-Z}_{model,i}^{Re}}{Z_{exp,i}^{Re}}\right)}^{2}+{\left(\frac{Z_{exp,i}^{Im}-Z_{model,i}^{Im}}{Z_{exp,i}^{Im}}\right)}^{2}$$

In both cases, χ^2^ values of 1.99 and 1.35 were obtained, indicating that the model provides a satisfactory fit to the experimental data. The values of the Q and α parameters exhibit only minor changes upon the addition of glucose compared to the blank solution, whereas the charge transfer resistance decreases significantly from 820 to 92 Ω. Such a decrease in charge transfer resistance in the presence of glucose clearly indicates an increased rate of redox reactions occurring on the sensor surface during glucose oxidation.

The impedance exhibits a strong dependence on the presence of glucose at a fixed measurement frequency, a property that can be exploited for the development of an AC-based sensor. As shown in Figure 10, the real part of the impedance, Re(Z), at a frequency of 0.1 Hz decreases by a factor of 3.7—from 493 Ω to 132 Ω—upon the addition of 1 mM glucose to the electrolyte. The imaginary part of the impedance, Im(Z), at a frequency of 1 Hz is particularly sensitive to glucose, decreasing more than ninefold—from 187 Ω to 20.7 Ω—as the glucose concentration increases from 0 to 1 mM.

**Figure 11:**
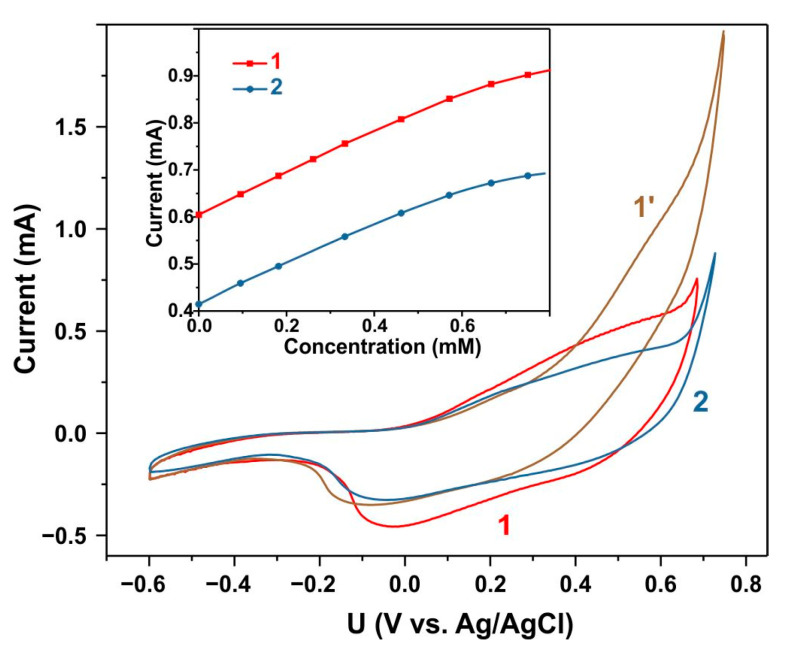
CV curves of the activated sample recorded in a 0.1 M KOH electrolyte at a scan rate of 100 mV s^−1^. Curves 1 and 1′ were measured in an electrolyte without glucose and with a glucose concentration of 1 mM, respectively. Curve 2 was recorded after 24 h in an electrolyte without glucose.

Sensor stability is a crucial performance characteristic. The synthesized material demonstrated high reproducibility of its sensory properties when stored under ambient conditions. The activated material demonstrated stability when stored in a 0.1 M KOH electrolyte. Figure 11 presents the CV characteristics of the activated sample in a blank electrolyte (curve 1) and in an electrolyte containing 1 mM glucose (curve 1′). The inset in Figure 11 illustrates the dependence of the current at a potential of 0.6 V vs. Ag/AgCl (curve 1). The sample was then returned to a blank electrolyte, and curve 2 in Figure 11 represents its CV characteristics after 24 h of immersion in the electrolyte. Although the current decreased slightly, the sensitivity remained unchanged (curve 2 in the inset of Figure 11).

**Figure 12:**
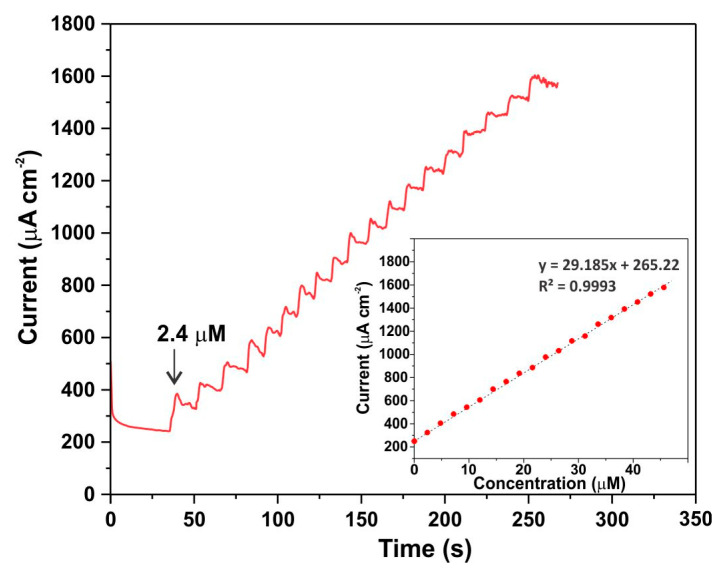
The amperometric curve obtained in 0.1 M KOH electrolyte; glucose with a concentration of 1 mM was added in portions of 100 µL. The insert shows the calibration curve for the amperometric determination of glucose.

Figure 12 illustrates the amperometric determination of glucose at an applied potential of 0.5 V vs. Ag/AgCl, where 0.1 mL of 1 mM glucose solution was sequentially added to 40 mL of 0.1 M KOH. With each addition of glucose, the current increased proportionally, exhibiting a linear relationship with the glucose concentration. The 90% response time, characterizing the sensor’s reaction time, did not exceed 10 s.

**Figure 13:**
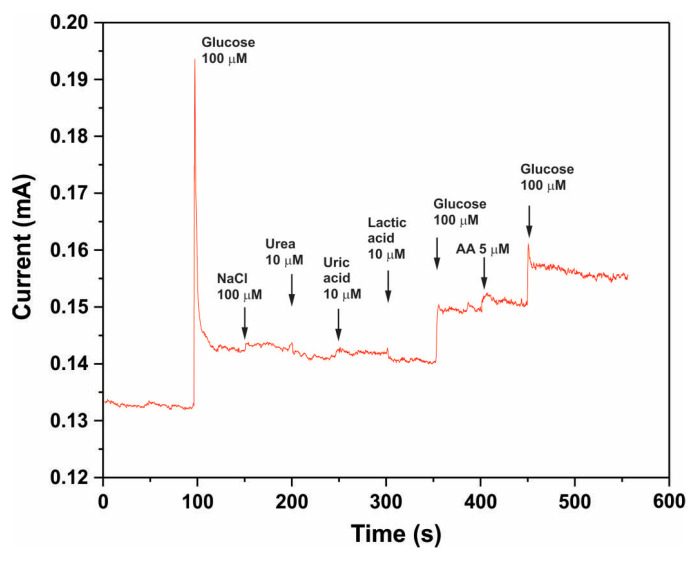
Interference testing of the activated electrode in a 0.1 M KOH solution at a potential of 0.6 V vs. Ag/AgCl with 100 µM glucose and interfering species, including NaCl (100 µM), urea (10 µM), uric acid (10 µM), lactic acid (10 µM), and ascorbic acid (5 µM).

Selectivity was evaluated through amperometric measurements by adding a specific amount of glucose along with interfering species such as NaCl, urea, uric acid, lactic acid, and ascorbic acid. Figure 13 shows the amperometric responses of the activated electrode in a 0.1 M KOH solution at a potential of 0.6 V vs. Ag/AgCl. The sensor exhibited only a slight response to these interfering species, demonstrating the high selectivity of the resulting Co_3_O_4_ for glucose detection in their presence.

Thus, the resulting Co_3_O_4_ electrode demonstrates a strong response to glucose. Cobalt hydroxycarbonate synthesized via the chemical bath deposition method is transformed into cobalt oxide hydroxide during activation under alkaline conditions. The positive potential applied to the electrode during glucose detection facilitates the formation of Co^4+^ states [2,37], which possess strong oxidizing properties. The formation of these states occurs by the following reactions:

Co_3_O_4_ + OH^−^ + H_2_O → 3CoOOH + e^−^, (2)

CoOOH + OH^−^ → CoO_2_ + H_2_O + e^−^. (3)

Glucose oxidation occurs through the following reaction:

2CoO_2_ + C_6_H_12_O_6_ (glucose) ↔ 2CoOOH + C_6_H_10_O_6_ (gluconolactone). (4)

This process is driven by the strong oxidizing ability of CoO_2_. The oxidation process is described by the following equations [2]:

Co^3+^ → Co^4+^ + e^−^ (5)

2Co^4+^ + C_6_H_12_O_6_ (glucose) → 2Co^3+^ + C_6_H_10_O_6_ (gluconolactone) + 2H^+^. (6)

The high sensitivity of the resulting sensor is likely attributable to the high dispersion of the material achieved through the applied synthesis method and the use of low synthesis temperatures. Table 1 provides a comparison of the characteristics of several cobalt-based non-enzymatic glucose sensors reported in the recent literature. The characteristics of the sensor developed in this study are comparable to those of other non-enzymatic glucose sensors. However, the proposed method offers advantages in its simplicity of hardware design, resource efficiency, and use of inexpensive precursors.

## 4. Conclusions

In conclusion, a simple chemical bath deposition method is proposed for synthesizing cobalt hydroxycarbonate, which transforms into a Co_3_O_4_ nanoparticle structure through electrochemical activation, enabling the creation of a non-enzymatic glucose sensor. The Co_3_O_4_ sensor demonstrates a detection limit of 5 µM and a glucose sensitivity of 33,250 µA mM^−1^cm^−2^. The sensitivity shows a linear dependence on the glucose concentration in the range of 0 to 0.5 mM and remains high up to 1 mM. The synthesized material exhibits good stability in its sensory properties. The sensitivity of the synthesized material remains stable for over 12 months when stored under ambient conditions. Additionally, the synthesis process does not require high temperatures, expensive precursors, or lengthy and complex procedures, making the material highly efficient and promising for scalability. The sensor demonstrates good resistance to several common interfering substances. However, it should be noted that its compatibility with real biological matrices—such as blood, serum, or interstitial fluid—has not yet been experimentally validated. Further investigation of the sensor’s stability and accuracy in complex biological environments appears promising, particularly using the electrochemical activation method developed in the present study.

## Figures and Tables

**Table 1:** A comparison of the characteristics of the developed sensor with those of non-enzymatic electrodes based on cobalt-containing materials.

Glucose Sensor	Linear Range (mM)	Sensitivity (µA × mM^−1^ × cm^−2^)	Detection Limit (μM)	Reference
ZnO/Co_3_O_4_/rGO composite	0.015–10 mM	1551.38	0.043	[27]
Co_2_P nanoparticles on carbon fiber	0.04–0.15 mM	409	0.97	[28]
Co(OH)_2_ nanosheet on carbon cloth	0.001–5.45 mM	6759	0.32	[29]
Co_3_O_4_ nanobooks	0–6 mM	1068.85	7.94	[38]
Co_3_O_4_ nanocubes	0.05 μM to 7.44 mM	19.3	0.01	[39]
Co_3_O_4_ nanowires	1 μM to 0.73 mM	26,170	0.56	[40]
Co_3_O_4_ nanoparticles	up to 3mM	2495.79	0.0093	[41]
Co_3_O_4_/rGO nanohybrid		82	50	[42]
Co_3_O_4_ Nanoneedles	1 μM–0.337 mM	4570	0.91	[43]
Co_3_O_4_ nanosheets	2–3.420 μM	745.26	0.65	[44]
Co_3_O_4_ microspheres	0.005–12 mM	1449	0.91	[45]
hierarchical Co_3_O_4_ architecture	0.53–1900 μM	839.3	0.08	[46]
Co_3_O_4_/Ni heterostructure	0–4 mM	13,855	1	[47]
RGO–Co_3_O_4_	1 µM–500 µM	1315	0.4	[48]
SS-Co_3_O_4_	0.04–4.85 mM	669	0.31	[49]
Co_3_O_4_ nanoparticles	up to 0.5 mM	33,245	5	This work

## Data Availability

Data is contained within the article.
